# Genome-Wide Association Study to Identify Genes Related to Renal Mercury Concentrations in Mice

**DOI:** 10.1289/ehp.1409284

**Published:** 2016-03-04

**Authors:** Hammoudi Alkaissi, Jimmy Ekstrand, Aksa Jawad, Jesper Bo Nielsen, Said Havarinasab, Peter Soderkvist, Per Hultman

**Affiliations:** 1 Molecular and Immunological Pathology, Department of Clinical Pathology and Clinical Genetics; Department of Clinical and Experimental Medicine, Linköping University, Linköping, Sweden; 2 Division of Cell Biology, Department of Clinical and Experimental Medicine, Linköping University, Linköping, Sweden; 3 Research Unit for General Practice, Institute of Public Health, University of Southern Denmark, Odense C, Denmark

## Abstract

**Background:**

Following human mercury (Hg) exposure, the metal accumulates in considerable concentrations in kidney, liver, and brain. Although the toxicokinetics of Hg have been studied extensively, factors responsible for interindividual variation in humans are largely unknown. Differences in accumulation of renal Hg between inbred mouse strains suggest a genetic interstrain variation regulating retention or/and excretion of Hg. A.SW, DBA/2 and BALB/C mouse strains accumulate higher amounts of Hg than B10.S.

**Objectives:**

We aimed to find candidate genes associated with regulation of renal Hg concentrations.

**Methods:**

A.SW, B10.S and their F1 and F2 offspring were exposed for 6 weeks to 2.0 mg Hg/L drinking water. Genotyping with microsatellites was conducted on 84 F2 mice for genome-wide scanning with ion pair reverse-phase high-performance liquid chromatography (IP RP HPLC). Quantitative trait loci (QTL) were established. Denaturing HPLC was used to detect single nucleotide polymorphisms for haplotyping and fine mapping in 184 and 32 F2 mice, respectively. Candidate genes (Pprc1, Btrc and Nfkb2) verified by fine mapping and QTL were further investigated by real-time polymerase chain reaction. Genes enhanced by Pprc1 (Nrf1 and Nrf2) were included for gene expression analysis.

**Results:**

Renal Hg concentrations differed significantly between A.SW and B10.S mice and between males and females within each strain. QTL analysis showed a peak logarithm of odds ratio score 5.78 on chromosome 19 (p = 0.002). Haplotype and fine mapping associated the Hg accumulation with Pprc1, which encodes PGC-1-related coactivator (PRC), a coactivator for proteins involved in detoxification. Pprc1 and two genes coactivated by Pprc1 (Nrf1 and Nrf2) had significantly lower gene expression in the A.SW strain than in the B10.S strain.

**Conclusions:**

This study supports Pprc1 as a key regulator for renal Hg excretion.

**Citation:**

Alkaissi H, Ekstrand J, Jawad A, Nielsen JB, Havarinasab S, Soderkvist P, Hultman P. 2016. Genome-wide association study to identify genes related to renal mercury concentrations in mice. Environ Health Perspect 124:920–926; http://dx.doi.org/10.1289/ehp.1409284

## Introduction

Mercury (Hg) is a toxic metallic element that contaminates the environment through both anthropogenic and nonanthropogenic sources (Tchounwou et al. 2003). The toxicological profile and metabolic fate of Hg in humans and animals depend on form, dose, age and exposure route (Clarkson and Magos 2006; Hultman and Nielsen 2001). Mercury exists mainly in three forms; elemental Hg (Hg^0^), inorganic Hg (Hg^2+^) and organic Hg (methyl- and ethyl-Hg) (Clarkson et al. 2007). The European Scientific Committee on Health and Environmental Risks (SCHER) determined dental amalgams as the dominant source of Hg^0^ in the general population by estimating the average daily intake and retention of total Hg and Hg compounds (SCHER 2007). Exposure to methyl-Hg (MeHg) from fish consumption has been a concern for decades [U.S. Environmental Protection Agency (EPA) 2014], and some groups have raised concerns about thimerosal (ethyl-Hg) in vaccines (U.S. EPA 2014). Hg^0^ (Halbach et al. 2008) and organic Hg (Clarkson et al. 2007) are transformed into Hg^2+^ in humans and animals at different rates and in different manners.

The thiol-containing protein glutathione (GSH) binds Hg to form GSH-Hg complexes (Lee et al. 2001; Schläwicke Engström et al. 2008; Zalups 2000) and is the primary form in which Hg is transported out of cells (Clarkson et al. 2007). Regulatory pathways of accumulation and excretion have not been fully elucidated (Bridges et al. 2014; Zalups 2000). GSH conjugates are transported into proximal tubular cells via organic anion transporters 1 and 3 (Oat1 and Oat3) (Hazelhoff et al. 2012) and are subsequently transported into the urine by multidrug resistance–tolerated proteins (MRPs) (Bridges et al. 2008a, 2011). Polymorphisms in the human *ABCC2* gene, which encodes MRP2, are associated with variations in urinary excretion of Hg^2+^ in populations exposed to Hg^0^ vapor from gold mining (Engström et al. 2013).

MRPs are regulated by nuclear factor-erythroid 2-related factor 2 (*Nrf2*). *Nrf2-*deficient mice exposed to methyl-Hg have increased Hg levels in brain and liver compared with wild type mice (Toyama et al. 2007). GSH is also controlled by the transcription factor nuclear respiratory factor 1 (*Nrf1*) in rats (Yang et al. 2005). Hepatocytes from *Nrf1* and *Nrf2* knockout mice exhibit reduced GSH levels (Chen et al. 2003; Kwong et al. 1999).

**Figure 1:**
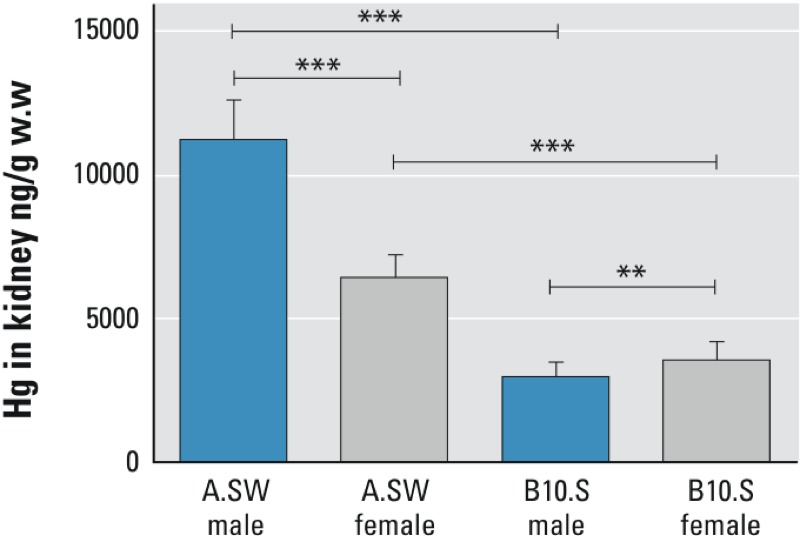
Kidney mercury concentrations. Mercury (Hg) deposition in kidneys of male and female A.SW and B10.S mice exposed to 2 mg Hg/L drinking water for 6 weeks. Data obtained from previous study (Ekstrand et al. 2010). Figure is presented as mean ± SD, **p = 0.0041, ***p < 0.0001 (Welch’s test).

Accumulation of renal Hg^2+^ has been reported to vary by sex in humans (Akesson et al. 1991) and rats (Thomas et al. 1987) and between mouse strains (Nielsen and Hultman 2002). In our previous study, which compared the two mouse strains A.SW and B10.S (Figure 1), A.SW mice accumulated more Hg than did B10.S mice. In terms of sex, male A.SW mice showed significantly greater accumulation of Hg than females of this strain, whereas B10.S mice showed the opposite trend (Ekstrand et al. 2010). Renal Hg measurement data from Ekstrand et al. (2010) were used to find candidate genes associated with regulation of renal Hg^2+^ accumulation in mice. We identified a chromosomal region on chromosome 19 in which the gene *Pprc1* (peroxisome proliferator-activated receptor gamma, coactivator-related) [PGC-1-related coactivator (PRC)] is a potential key regulator of renal Hg accumulation and elimination.

## Materials and Methods

### Mice

Male and female A.SW mice (*n* = 18 and 17, respectively) were obtained from Taconic, and B10.S mice (*n* = 20 and 23, respectively) were obtained from the Jackson Laboratory. F1-hybrids (*n* = 19 males, 20 females) were derived by crossing female A.SW and male B10.S mice. F2-hybrids (*n* = 154 males, 180 females) were obtained by crossing F1-hybrids. Mice were housed at the Animal Facilities, Linköping University, Sweden and were kept under a controlled environment with 5–10 mice/cage. The mice were offered standard mouse pellets (CRME rodent, Special Diets services) and drinking water *ad libitum*. Studies were approved by the Laboratory Animal Ethics Committee, Linköping, Sweden, and all mice were treated humanely with regard to alleviating any suffering.

### Exposure and Design

All mice (A.SW, B10.S, F1, and F2) were given 2.7 mg HgCl_2_/L (Fluka) in drinking water (2.0 mg Hg/L) at age 8–10 weeks, for 6 weeks before sacrifice (age 14–16 weeks). No mice were exposed during pregnancy. HgCl_2_ was mixed with ^203^Hg isotope, and 1 mL drinking water contained 35,000–45,000 counts per minute. Radioactivity of the left kidney, obtained after sacrifice, was measured using a gamma counter (Perkin Elmer, 2470 Wizard) and used to quantify renal Hg accumulation.

Renal Hg concentration in F2 mice (*n* = 334) was classified as “high” (> 5,836 ng/g wet weight, the highest concentration in F1 mice), “low” (< 2,990 ng/g wet weight, the lowest concentration in F1 mice) and “intermediate” (2,990–5,836 ng/g wet weight, the range of concentrations observed in F1 mice).

The genome-wide scan was performed on 28 F2 mice selected at random from each group using the randomized function RANDBETWEEN in Microsoft Excel (McFedries 2010), for a total of 84 mice (44 male and 40 female). Haplotyping was performed on 334 F2 mice to narrow down the quantitative trait loci (QTL) region. Fine gene mapping on 32 F2 mice was performed on a haplotype for detection of candidate genes. Gene expression of candidate genes and genes enhanced by candidate genes was performed on 7 male and 7 female A.SW and B10.S mice (28 mice total), as described in detail below.

### Single Nucleotide Polymorphisms, Microsatellites, and Primer Design

Single nucleotide polymorphisms (SNPs) and microsatellites were identified using the Ensembl (Flicek et al. 2014) and Mouse Genome Informatics (MGI) databases (Blake et al. 2014). NCBI/Primer-BLAST was used to design primers (Ye et al. 2012). The sex chromosome was excluded from the genome-wide association study (GWAS) because no microsatellites on this chromosome differed between A.SW and B10.S mice (data not shown). Accession numbers were obtained from the UniProt database (UniProt Consortium 2015).

### DNA Extraction and Genotyping

DNA isolated from tail, spleen, or kidney was extracted using the Wizard® SV Genomic DNA Purification System (Promega). These tissues were used to achieve the required amount and concentration of DNA. The quantity and purity of the DNA were measured with a NanoDrop ND-1000 spectrophotometer (Thermo Fisher Scientific). The measured spectrophotometric absorbance A260/A280 ratio was 1.8–2.0, and samples were diluted to 20 ng/μL. Samples were genotyped using microsatellites (see Table S1) or designed primers covering SNPs (see Table S2) (Invitrogen, Life Technologies). The polymerase chain reaction (PCR) conditions were 30 sec at 94°C, 1 min annealing (55–63°C), and 1 min at 72°C for 35 cycles. DNA amplification was verified by gel electrophoresis.

### Ion Pair Reverse-Phase High-Performance Liquid Chromatography

Microsatellites between 2 and 10 bp were detected using ion pair reverse-phase high-performance liquid chromatography (IP RP HPLC) on a Transgenomic WAVE system (Transgenomic). The mobile phase consisted of 0.1 M triethylammonium acetate (TEAA; Applied Biosystems) (Solvent A) and 0.1 M TEAA–25% acetonitrile (ACN; EM Science) (Solvent B). The percentage of Solvent B, the column temperature, and the flow rate (milliliters per minute) were optimized for each microsatellite.

The detection of SNPs from PCR amplicons was analyzed by denaturing HPLC (dHPLC) using a Transgenomic WAVE system. The PCR products for F2 mice were pooled according to strain (A.SW or B10.S) and were denatured by heating at 96°C for 5 min followed by gradual cooling to 25°C for 30 min. The PCR products were loaded on a DNAsep column (Transgenomic) and eluted using a linear ACN gradient in a 0.1 M TEAA buffer (pH 7) with a constant flow rate of 0.9 mL/min. The gradient start and end points were optimized according to amplicon size. The melting temperatures selected for optimal separation of the amplified DNA products were calculated using WAVEMAKER™ software, v.3.3.3 (Transgenomic).

### Linkage Analysis

Linkage analysis was performed to evaluate candidate genes associated with renal Hg accumulation. QTL were identified based on logarithm of odds (LOD) score profiles derived from a genome-wide single-QTL scan by Haley-Knott regression (Knott and Haley 1992) with a Hidden Markov model (HMM) using R/qtl software (v.2.15.3) (Broman et al. 2003). Regression was based on data from 84 F2 offspring for 96 microsatellites covering 19 autosomes with an average spacing of 20 cM (see Table S1). Genotype data were 99.7% complete. The genome-wide significance threshold was calculated based on 10,000 permutation replicates. Additional microsatellites were used to narrow the region with haplotype analysis in which a QTL was found (see Table S3). All F2 offspring were genotyped in the QTL region, and haplotypes were identified by comparing the genotype of F2 mice with the genotypes of A.SW and B10.S mice. Fine mapping was based on genotyping A.SW, B10.S, and F2 mice with SNP markers covering the haplotype, followed by locating additional QTL on F2 mice.

### Sanger Sequencing

Sequencing of SNPs in *Lbx1* (P52955) and *Tlx1* (P43345) was performed to clarify whether SNPs in background strains A (for A.SW) and C57BL/6 (for B10.S), according to the Ensembl and MGI databases, were present in A.SW and B10.S mice because dHPLC did not show any SNPs. PCR primers covering exons in which SNPs were predicted, including exon/intron borders, were used to generate PCR products (see Table S4). Residual nucleotides were removed using ExoProStar 1-Step (GE Healthcare), and the PCR products were sequenced according to a standard protocol for fluorescently labeled dideoxynucleotides (Applied Biosystems, Life Technologies) and separated on a capillary electrophoresis instrument (ABI 3500, Life Technologies).

### RNA Extraction, cDNA Reverse Transcription, and Real-Time PCR Analysis

Total RNA was extracted from kidneys using an RNeasy Mini Kit (Qiagen) according to the manufacturer’s instructions. The quantity and purity of the RNA were measured using a NanoDrop ND-1000 spectrophotometer at an A260/A280 value of 1.8–2.0, and the RNA was diluted to 20 ng/μL. cDNA was synthesized by reverse transcription of 0.2 μg total RNA using a High-Capacity cDNA Archive Kit (Applied Biosystems). Analysis was performed in duplicate using the Applied Biosystems 7500 Fast Real-Time PCR System with Applied Biosystems TaqMan® Gene Expression Assays (Applied BioSystems). Target gene expression for *Pprc1* (Q6NZN1), *Nrf1*, *Nrf2, Btrc* (Q3ULA2) and *Nfkb2* (Q9WTK5) was measured with FAM (6-carboxyfluorescein) reporter dye–labeled probes (see Table S5). *Pprc1, Btrc* and *Nfkb2* were selected because fine mapping and QTL analysis revealed them as candidate genes. *Pprc1* acts as a co-activator for *Nrf1* (Andersson and Scarpulla 2001) and *Nrf2* [via CREB - cAMP Responsive Element Binding protein (Andersson and Scarpulla 2001; Katoh et al. 2001)]; therefore, it was also analyzed. Ten genes were evaluated as potential endogenous controls (see Table S6). The criterion for selection of housekeeping genes was based on minimal fluctuation of Ct values assumed to be independent of Hg exposure between samples. *Gapdh* and *Ppia* were selected as endogenous controls after Ct value determination using normfinder (Andersen et al. 2004). Ct variation of *Gapdh* and *Ppia* was < 1 Ct. The geometric means of *Gapdh* and *Ppia* in each group were used as endogenous controls (see Table S7). The results are presented as relative transcription using the comparative Ct method. ΔCt1 was calculated for each of the target genes in every mouse by subtracting the endogenous control (the geometric means for *Gapdh* and *Ppia*) for each sample. ΔCt2 was calculated by subtracting reference genes in untreated F1 mice (because parental strains were examined). ΔΔCt was calculated by subtracting ΔCt2 from ΔCt1, and finally, relative quantification was calculated as 2^–ΔΔCt^.

### Statistical Analysis

Gene expression and genotype versus phenotype data were tested for normality using the D’Agostino–Pearson omnibus normality test, which computes a *p*-value for the combination of the coefficients of skewness and kurtosis (D’Agostino 1986). Data that did not pass the normality test are presented as medians ± interquartile ranges, and comparisons between two groups were performed using the Mann–Whitney U-test. Data that did pass the normality test are presented as the mean ± SD, and comparison between two groups was performed using Welch’s *t*-test. Differences with *p* < 0.05 were considered significant.

## Results

### Characterization of the B10.S and A.SW Strains

**Figure 2:**
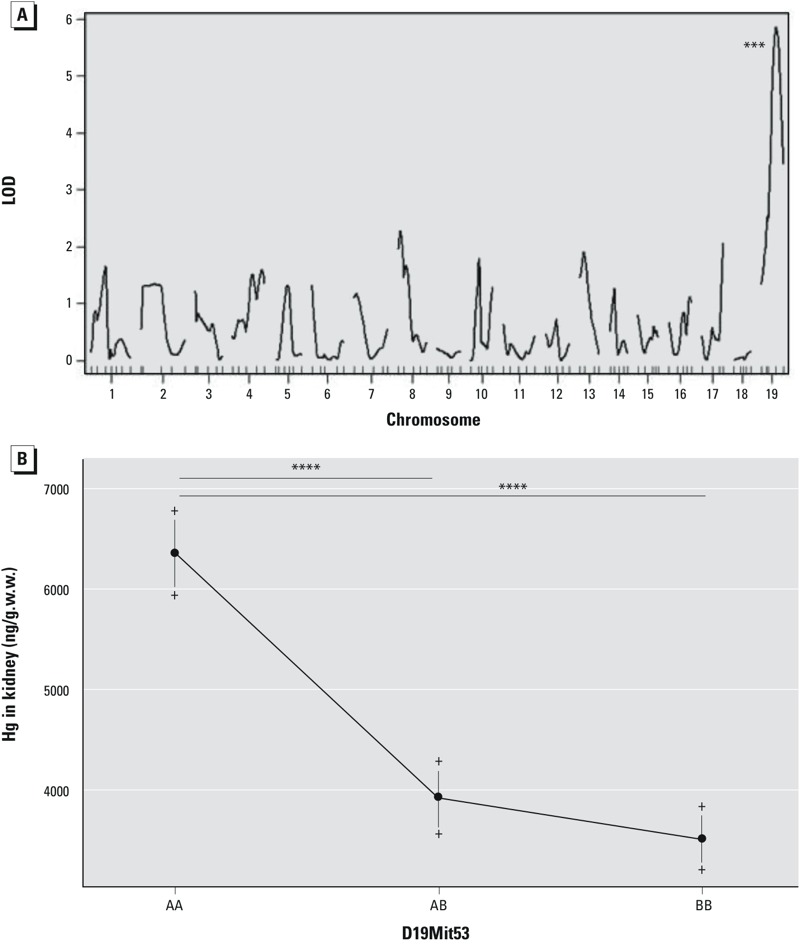
Quantitative trait loci on autosomes and effect plot. (*A*) Genome-wide scan (*n *= 44 male, 40 female F2 mice) on autosomes was performed to identify quantitative trait loci (QTL) associated with Hg accumulation in kidney. Logarithm of odds (LOD) scores (*y*-axis) indicate a high association with microsatellite D19Mit53 on chromosome 19; LOD score = 5.78, ***p = 0.0002. (*B*) Mean ± SD renal Hg concentration (ng/g wet weight) according to D19Mit53 genotype. AA, homozygous for the A.SW allele; BB, homozygous for the B10.S allele; AB, heterozygote; ****p < 0.0001 (Mann–Whitney test).

***Genetic linkage.*** A highly significant (*p* = 0.0002) QTL, located at 38.46 cM (D19Mit53) on chromosome 19, had a LOD score of 5.78. QTLs were also detected on chromosomes 8 (12.59 cM), 13 (27.48 cM) and 17 (55.48 cM), all of which had LOD scores ≥ 2 (Figure 2A). Renal Hg accumulation was significantly higher (*p* < 0.0001) in F2 mice that were homozygous for the A.SW allele of D19Mit53 than in heterozygotes or in mice that were homozygous for the B10.S allele, suggesting autosomal recessive inheritance (Figure 2B).

**Figure 3:**
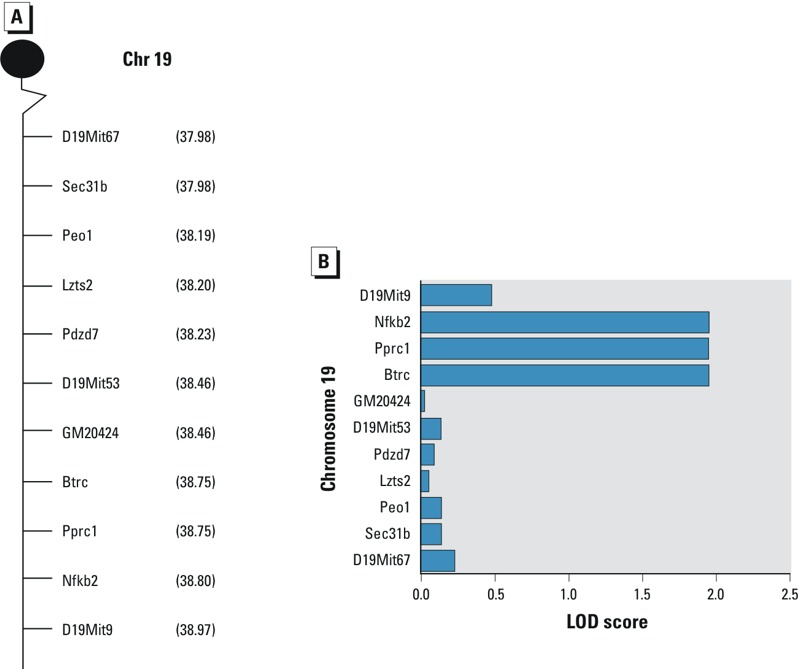
Fine mapping and QTL. (*A*) Markers used for fine mapping in haplotype position between 37.98 and 38.97 cM on chromosome 19 were homozygous for the A.SW allele on D19Mit53; 184 F2 mice were analyzed. (*B*) QTL associated with Hg accumulation in kidney based on fine mapping results on chromosome 19, on 32 F2 offspring homozygous for A.SW on D19Mit53. *Btrc, Pprc1* and *Nfkb2* all had a LOD score of 1.94.

Haplotype analysis of D19Mit53 (38.46 cM) indicated that 32 of 184 F2 mice were homozygous for the A.SW allele. Additional genotyping with 20 microsatellites (see Table S3), spaced between 20.18 and 56.28 cM, identified a DNA block between microsatellites D19Mit67 (37.98 cM) and D19Mit9 (38.97 cM) in which the 32 F2 homozygous mice were further analyzed with fine mapping. Regression was based on 11 markers consisting of 3 microsatellites and 8 SNPs (Figure 3A) because these markers were polymorphic between background strains and between A.SW and B10.S strains. *Lbx1*, *Tlx1*, and *Poll* genes within this haplotype are polymorphic between the background strains (Flicek et al. 2014; Blake et al. 2014) but showed no difference between A.SW and B10.S strains (data not shown) and were therefore excluded. Other genes within this haplotype were not analyzed because they were not polymorphic between the background strains.

Fine mapping narrowed the region to 19:45630547–19:46384795 with a LOD score of 1.94 (Figure 3B). SNP analysis revealed three genes segregated between background strains; *Btrc*, *Pprc1*, and *Nfkb2*. All of the SNPs for *Btrc* and *Nfkb2* were located in untranslated regions: *Btrc* had 12 SNPs (5´-UTR) and *Nfkb2* had 1 SNP (3´-UTR) (data not shown). *Pprc1* had 7 nonsynonymous SNPs, denoted SNP^1–7^, all of which resided on exon 5 (see Table S8).

**Figure 4:**
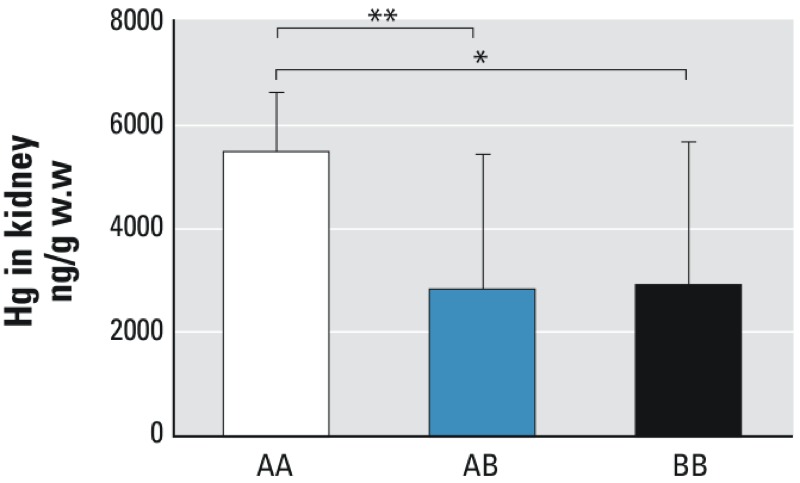
SNP genotype in *Pprc1.* Genotype data of F2 offspring on two SNPs, rs30400427 (A.SW, guanine; B10.S, adenine) and rs30815571 (A.SW, adenine; B10.S, guanine) in *Pprc1.* The *x*-axis shows homozygous genotypes for A.SW (AA) and B10.S (BB) and a heterozygous genotype (AB). Graph is presented as median ± interquartile range, *p = 0.0299; **p = 0.0018 (Mann–Whitney test).

F2 mice that were homozygous for the A.SW allele (AA) of two SNPs (rs30400427 and rs30815571) in *Pprc1* showed significantly higher Hg accumulation than heterozygous (AB) (*p* = 0.0018) and homozygous B10.S (BB) (*p* = 0.0299) mice (Figure 4). Hg accumulation was not significantly different between *Pprc1* BB and AB F2 mice. Mice that were homozygous for the A.SW alleles of *Btrc* and *Nfkb2* also had higher renal Hg accumulation than heterozygotes (AB) or homozygous (BB) mice (data not shown). Hg accumulation was not significantly different between F2 mice that were BB and AB for *Btrc* and *Nfkb2* (data not shown).

Comparison of SNP^1–6^ on *Pprc1* among 15 mammals (mouse, rat, human, rabbit, marmoset, vervet-AGM, olive baboon, gorilla, orangutan, cow, sheep, pig, dog, cat, and horse) was performed using the Ensembl database (see Figure S1) (Flicek et al. 2014). SNP^1^ in mouse was not located in a conserved region. SNP^2^ was located in a conserved region in 13 mammals, and SNP^3^ was conserved in all 15 mammals. SNP^4^ was in a conserved region in 7 mammals. SNP^5^ and SNP^6^ were conserved between mouse and rat only.

Comparison of SNP^7^ (rs30352970) in *Pprc1* among 36 mammals (mouse included) and 3 species of birds was performed using the Ensembl database (see Figure S2) (Flicek et al. 2014). SNP^7^ was conserved in 33 of the mammalian species and in all 3 bird species. SNP^7^ has the codon GGT (glycine) in the B10.S strain and AGT (serine) in the A.SW strain. The conserved region of the amino acid sequences was analyzed using Clustal X (version 2.1) multiple sequence alignment software, which aligns sets of amino acid sequences (Larkin et al. 2007). Sequence alignment was performed on 15 mammalian species (mouse, rat, human, rabbit, marmoset, vervet-AGM, olive baboon, gorilla, orangutan, cow, sheep, pig, dog, cat, and horse) against which the Ensembl database has run a nucleotide alignment (see Figure S3). Fourteen of the sequences coded for the same amino acid, glycine, as the B10.S strain. Ensembl data indicated that the SNPs in *Btrc* and *Nfkb2* were located in non-conserved regions (Flicek et al. 2014).

***Gene expression.*** Differences in renal mRNA expression of *Pprc1*, *Btrc*, *Nrf1*, and *Nrf2* between A.SW and B10.S were examined (Table 1). *Pprc1* acts as a co-activator for *Nrf1* (Andersson and Scarpulla 2001) and *Nrf2* [via CREB (Andersson and Scarpulla 2001; Katoh et al. 2001)] and, therefore, was also analyzed. For males and females combined, *Pprc1*, *Nrf1*, and *Nrf2* mRNA expression was approximately 5 times higher in B10.S mice than in A.SW mice (all *p* < 0.0001). Both male and female B10.S mice showed significantly higher expression of *Pprc1* (*p* = 0.0014 and *p* = 0.0056, respectively), *Nrf1* (*p* = 0.015 and *p* = 0.0083, respectively) and *Nrf2* (*p* = 0.0045 and *p* = 0.0049, respectively) than male and female A.SW mice. When comparing sexes within each strain, the mRNA expression of *Pprc1* in A.SW male mice was ~9 times higher than in female A.SW mice (*p* = 0.0103). *Nrf1* mRNA expression was ~12 times higher in male than in female mice (*p* = 0.0001), and *Nrf2* was ~9 times higher in male compared to female mice (*p* = 0.0133). mRNA expression of *Pprc1*, *Nrf1* and *Nrf2* in B10.S mice showed no significant differences between males and females. *Btrc* mRNA expression differed significantly (*p* = 0.0437) between male and female B10.S mice only, whereas the expression of *Nfkb2* was significantly higher in male A.SW mice than in male B10.S mice (*p* = 0.0299).

**Table 1:** Fold difference in renal mRNA expression (mean ± SD) following 6 weeks of Hg^2+^ exposure.

Strain and sex	Number of mice	*Pprc1*	*Nrf1*	*Nrf2*	*Btrc*	*Nfkb2*
A.SW
Both	14	0.93 ± 0.71^†^	0.54 ± 0.46^†^	0.71 ± 0.61^†^	3.94 ± 2.68	22.6 ± 7.97
Male	7	1.37 ± 0.50*^†^	1.06 ± 0.14*^†^	1.38 ± 0.34*^†^	5.01 ± 3.14	24.53 ± 8.78^†^
Female	7	0.15 ± 0.09*^†^	0.09 ± 0.04*^†^	0.15 ± 0.08*^†^	2.88 ± 1.50	20.68 ± 6.53
B10.S
Both	14	4.29 ± 1.33^†^	3.04 ± 1.44^†^	4.13 ± 1.52^†^	6.64 ± 3.84	14.58 ± 8.58
Male	7	3.99 ± 1.52^†^	2.40 ± 0.90^†^	3.43 ± 1.10^†^	9.19 ± 3.53*	10.56 ± 3.49^†^
Female	7	4.20 ± 1.5^†^	2.70 ± 1.05^†^	3.96 ± 1.33^†^	4.09 ± 1.98*	18.61 ± 10.13
Gene expression in kidney obtained from male and female A.SW and B10.S mice exposed to Hg^2+^ for 6 weeks. The mean ± SD of fold change is presented for each group. *Significant difference (*p* < 0.05) between sexes within a strain. ^†^Significant difference between strains (*p* = 0.0021, Welch’s test). *Gapdh* and *Ppia* were used as endogenous controls, fold change is relative to one unexposed F1 mouse (reference sample).						

## Discussion

**Figure 5:**
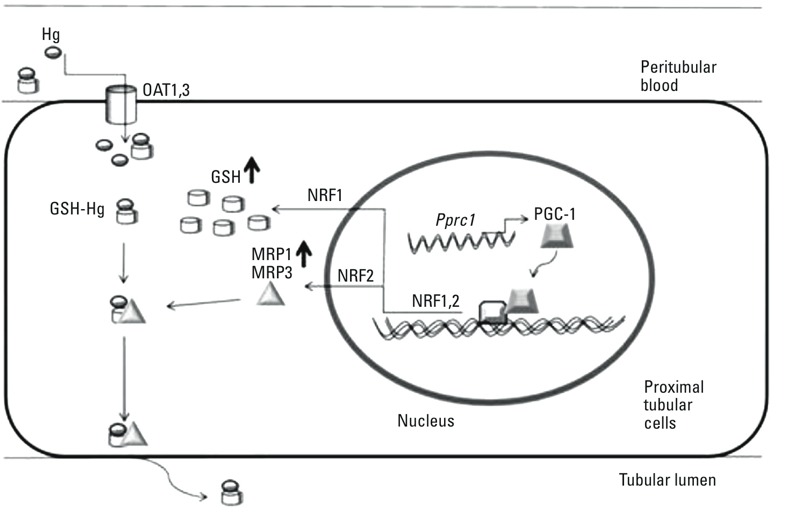
Hg excretion from kidney. Hypothetical conceptual model consistent with our findings. Mercury enters proximal tubular cells via OAT-1, OAT-3 transporter proteins (Bridges and Zalups 2005) already bound to sulfhydryl protein GSH or binds to the protein inside tubular cells. MRP1-3 export GSH-Hg complexes out of the cell into the tubular lumen and out with the urine (Bridges et al. 2008b; Toyama et al. 2007). *Pprc1* encodes the protein PGC-1-related coactivator, which acts as a coactivator for *Nrf1* and *Nrf2 *(via CREB)**transcription factors. *Nrf1* regulates production of GSH levels (Chen et al. 2003; Kwong et al. 1999; Yang et al. 2005), and* Nrf2* regulates production of MRP-1 and MRP-2 (Toyama et al. 2007).

Genome-wide genotyping, haplotyping, and fine mapping linked renal Hg accumulation to three genes with identical LOD scores of 1.94: *Btrc*, *Pprc1*, and *Nfkb2.* The *Pprc1* gene encodes the protein PRC (PGC-1-related coactivator), which is a member of the PGC-1 family. Its role is to regulate mitochondrial biogenesis in response to environmental signals (Lin et al. 2005). PRC is a coactivator for *Nrf1* (Andersson and Scarpulla 2001) and *Nrf2* [via CREB (Andersson and Scarpulla 2001; Katoh et al. 2001)]. Nrf1 enhances intracellular levels of GSH, which complexes with Hg, and Nrf2 increases MRP levels to stimulate the elimination of Hg-GSH (Bridges et al. 2008b, 2011) via proximal tubular cells into tubular lumen and out of the body (Figure 5) (Clarkson et al. 2007).

Between the A.SW and B10.S strains, we found seven SNPs in exon 5 of *Pprc1* with missense protein variants and amino acid exchanges that may alter gene expression and protein folding, function, and regulation (Hamosh et al. 2005). Analysis in the Ensembl database showed that SNP^7^ (rs30352970) was conserved in > 33 mammals (Flicek et al. 2014), which suggests that it provides a vital function for organisms (see Figure S2). A.SW has the codon AGT, which codes for serine, and B10.S has the codon GGT, which codes for glycine. When multiple sequence alignment was used to compare the conserved region of amino acids on rs30352970 in 15 mammals, 14 of the mammals had the same amino acid (glycine), as the B10.S strain (Larkin et al. 2007) (see Figure S3). Therefore, the substitution of serine for glycine (Gly1007Ser) may be responsible for the increased renal accumulation of Hg in A.SW mice compared with that in B10.S mice. The Ensembl data indicated that none of the SNPs in *Btrc* and *Nfkb2* were in conserved regions when a variety of mammalian species were compared (Flicek et al. 2014).

To understand the importance of variants of *Pprc1* in Hg accumulation, we compared reported renal Hg concentrations in four different mouse strains and nucleotide structure on exon 5 missense variants for *Pprc1.* Significant differences in renal Hg accumulation relative to B10.S mice have been reported for A.SW (Ekstrand et al. 2010; Griem et al. 1997; Nielsen and Hultman 1998), DBA/2 (Griem et al. 1997; Nielsen and Hultman 1998), and BALB/c (Tanaka-Kagawa et al. 1998) strains, which share the same alleles for *Pprc1* SNPs^1–7^ (Table 2), according to the Ensembl database (Flicek et al. 2014).

**Table 2:** *Pprc1* genotypes according to strain.

*Pprc1* SNP	B10.S	A.SW	DBA/2	BALB/c
rs30400427 SNP^1^	A	G	G	G
rs30815571 SNP^2^	G	A	A	A
rs30566249 SNP^3^	C	T	T	T
rs30507907 SNP^4^	T	C	C	C
rs30750332 SNP^5^	A	G	G	G
rs30360955 SNP^6^	C	T	T	T
rs30352970 SNP^7^	G	A	A	A
SNP, single nucleotide polymorphism. Nucleotide structure on exon 5, missense variants (SNP^1–7^) on *Pprc1* in B10.S, A.SW, DBA/2, and BALB/c strains. SNPs on B10.S and A.SW strains were confirmed with denaturing high-performance liquid chromatography. SNPs on DBA/2 and BALB/c strains were confirmed with Ensembl database (Flicek et al. 2014).				

Both male and female A.SW mice showed significantly lower (all *p* < 0.0001) mRNA expression of *Pprc1*, *Nrf1*, and *Nrf2* than male and female B10.S mice. Polymorphic variants in *Btrc* and *Nfkb2* were localized in the 5´-UTR and 3´-UTR. Regulatory elements in 5´-UTRs may influence the translation of downstream cistrons (Barrett et al. 2012). We did not find statistically significant differences in *Btrc* or *Nfkb2* mRNA between B10.S and A.SW mice, which suggests that the genetic differences do not influence gene expression. However, because fine mapping and QTL within the haplotype linked *Pprc1*, *Nfkb2*, and *Btrc* as possible candidate genes, SNPs on UTRs of *Nfkb2* and *Btrc* may be in linkage disequilibrium with SNPs on *Pprc1*. This linkage disequilibrium might generate a (high) possibility that *Pprc1* gene expression differences between A.SW and B10.S could be due to SNPs in UTRs of *Nfkb2* and *Btrc* instead of nonsynonymous SNPs in *Pprc1*.

Male and female B10.S mice had significantly higher expression of *Pprc1*, *Nrf1*, and *Nrf2* than did male and female A.SW mice. Our experimental setup mainly addressed strain differences; however, we did observe sex-related differences in A.SW mice. There was no significant difference in the expression of *Pprc1*, *Nrf1*, and *Nrf2* between male and female B10.S mice, in contrast to A.SW mice; in this strain, A.SW females had significantly lower expression of *Pprc1*, *Nrf1*, and *Nrf2* than did males. The difference was greater between the sexes of the A.SW strain (≤ 12 times) than that between the strains (5 times).

Several factors may explain the gene expression differences between the sexes and the strains; sex hormones, age, and duration of Hg exposure. The sex hormone estrogen upregulates transcription of PGC-1-related coactivator (PRC) in rats. Ovariectomized rats subcutaneously treated with 17β-estradiol showed increased expression of *Pprc1* in cerebral blood vessels compared with ovariectomized rats treated with placebo (Kemper et al. 2014). In our study, female A.SW mice showed a significant decrease of *Pprc1* expression compared with males. All mice were 8–10 weeks old before being exposed to Hg for 6 weeks, which may have affected estradiol levels. *Pprc1* expression showed no significant difference between sexes in the B10.S strain, which suggests that the genetic backgrounds of A.SW and B10.S differ in the estradiol gene or in genes regulating estradiol. Because the present study was performed on autosomes only, we cannot rule out genetic differences between strains on the sex chromosomes related to the regulation of sex factors on *Pprc1*. *Pprc1* expression may also vary according to the duration of Hg exposure. *Pprc1* expression was measured only once after 6 weeks of Hg exposure, and it is possible that expression may peak at an earlier or later point in time.

*Pprc1* may be a key regulator in the detoxification process of different forms of Hg and in different organs because inhaled Hg vapor is oxidized to Hg^2+^ in the blood, and Hg^2+^ can also be formed as part of the metabolism of organic Hg at different rates (Clarkson et al. 2007). GSH-Hg complexes and Nrfs have been identified in liver, kidney, and brain and appear to be the primary form in which Hg is transported out of cells (Clarkson et al. 2007; Toyama et al. 2007).

Some studies have reported that urinary Hg concentrations in humans correlate with numbers of dental amalgam surfaces; that is, people with more amalgam fillings showed higher urinary Hg concentrations (Barregard 2005; Bates 2006; Dutton et al. 2013). Other studies showed no significant correlation (Ganss et al. 2000; Vamnes et al. 2000). The human *PPRC1* gene has > 120 SNPs in exon 5 (Flicek et al. 2014), and at least one that shares the same position as rs30352970 in mouse is evolutionarily conserved. Genetic variation in *PPRC1* might modify associations between urinary Hg excretion and amalgam fillings in humans (Barregard 2005; Bates 2006; Maserejian et al. 2008).

## Conclusion

In conclusion, our findings suggest that *Pprc1* is a plausible candidate for a key regulator of renal Hg concentrations based on a genome-wide scan that linked renal Hg accumulation to chromosome 19 with a LOD score of 5.78 and fine mapping that identified a QTL on *Pprc1* with a LOD score of 1.94. Seven SNPs in *Pprc1* that give rise to different amino acids in A.SW and B10.S mice were also associated with renal Hg concentrations, including two SNPs (SNP^2^ and SNP^3^) that are in conserved regions in 13 mammalian species and one (SNP^7^, rs30352970) that is in a conserved region in 33 mammalian species and codes for the same amino acid in 14 mammalian species. In addition, mouse strains with high renal Hg concentrations share the same nucleotide sequence in *Pprc1*, and significant differences in gene expression between A.SW and B10.S strains were correlated with renal Hg accumulation. Gene expression of *Nrf1* and *Nrf2*, which *Pprc1* regulates, showed the same pattern as *Pprc1* expression, and is involved in the excretion of Hg.

## Supplemental Material

(1.3 MB) PDF
