# Supplementary material for: Genome-Wide Association Study to Identify Genes Related to Renal Mercury Concentrations in Mice
Source: Environ Health Perspect. 2016 Mar 4;124(7):920–6. doi: 10.1289/ehp.1409284 (PMC4937848; doi:10.1289/ehp.1409284)
Supplement: (1.3 MB) PDF [file ehp.1409284.s001.acco.pdf]

Environ Health Perspect
DOI: 10.1289/ehp.1409284

Note to readers with disabilities: *EHP* strives to ensure that all journal content is accessible to all readers. However, some figures and Supplemental Material published in *EHP* articles may not conform to 508 standards due to the complexity of the information being presented. If you need assistance accessing journal content, please contact ehp508@niehs.nih.gov. Our staff will work with you to assess and meet your accessibility needs within 3 working days.

Supplemental Material

Genome-Wide Association Study to Identify Genes Related to Renal Mercury Concentrations in Mice

Hammoudi Alkaissi, Jimmy Ekstrand, Aksa Jawad, Jesper Bo Nielsen, Said Havarinasab, Peter Soderkvist, and Per Hultman

Table of Contents

Table S1. Microsatellite markers used in the genome-wide scan
Table S2. Designed primers used for fine mapping
Table S3. Microsatellite markers used for haplotyping
Table S4. PCR primers amplified for Sanger Sequencing
Table S5. Gene targets for Real-Time PCR
Table S6. Potential housekeeping genes tested for Hg exposure
Table S7. Housekeeping genes selected for Hg exposure
Table S8. Codons and amino acids of SNPs on *Pprc1*
Figure S1. Conserved regions of SNPs 1-6
Figure S2. Conserved region of SNP 7.3
Figure S3. Amino acid alignment of SNP[7] in *Pprc1*

Table S1. Microsatellite markers used in the genome-wide scan

					Amplicon size	
**Markers**	**Chr**	**cM**	**Direction**	**Primer Pairs**	**Strain A/J**	**Strain C57Bl/6**
D1Mit430	1	10	**F**	TATTAATGTTGAAGCCAGAAGCC	124	114
			**R**	CTTTAATCATCTCTGTGGCAAGG		
D1Mit232	1	20.80	**F**	TTGTCCTCTGACCTTGCAGA	148	142
			**R**	TCCCCCTTCATTTCCTCTTT		
D1Mit132	1	43.10	**F**	TATTGTTTATGGAAATTGGACCC	162	148
			**R**	CATCTCTGAAGGAAAAAGTGCA		
D1Mit309	1	63.10	**F**	GTAGAGAATGATAAAGGACATCCTCC	156	127
			**R**	GGGAGGCTGAGATCTGTCAG		
D1Mit200	1	73.00	**F**	GCCATGTTCATGTACATAGGTAGG	297	205
			**R**	ATGGATGGATGGTTTTCCTG		
D1Mit111	1	92.30	**F**	ATTGCCTGACTCCAGTATTCTACC	188	170
			**R**	TTAGGTGTGTGAAAGACATTCCC		
D1Mit155	1	112.00	**F**	ATGCATGCATGCACACGT	216	252
			**R**	ACCGTGAAATGTTCACCCAT		
D2Mit117	2	5.00	**F**	CCCAAAGAACATACATCAATGTG	170	176
			**R**	TGGAGATGCATGTTTAAAACTCA		
D2Mit152	2	18.00	**F**	CACAGATCTTGTAAGACCACGTG	107	113
			**R**	TGCCATGAGTGTGGGACTAA		
D2Mit66	2	47.80	**F**	GTTGCACAGGCAATCAACC	278	256
			**R**	ATCTATCACTGGGGCTGTGC		
D2Mit260	2	83.00	**F**	ACATAGAAACAAGCATACATGCA	162	170
			**R**	CTGTGGTAAACTTTAAATAATGGTGG		
D2Mit148	2	105.00	**F**	GTTCTCTGATCTACGGGCATG	133	117
			**R**	TTCACTTCTACAAGTTCTACAAGTTCC		
D3Mit203	3	11.20	**F**	CTGAATCCTTATGTCCACTGAGG	138	154
			**R**	GGGCACCTGCATTCATGT		
D3Mit98	3	39.40	**F**	TTTGACCAACTCAGGTTCAGG	127	115
			**R**	GATGGCTGTGAACCAGTCAA		
D3Mit78	3	55.00	**F**	CATAAAGTGTCAAAACAACCAAGC	106	116
			**R**	TATGGGATCTCCCCACATGT		
D3Mit320	3	71.80	**F**	AATGAAATCTCACGAGAGGCA	108	120
			**R**	AAGCCAGGAGCAGAGTCAAG		
D3Mit19	3	87.60	**F**	CAGCCAGAGAGGAGCTGTCT	176	160
			**R**	GAACATTGGGGTGTTTGCTT		
D4Mit18	4	5.20	**F**	AATTAGCCCGGAGCTTGATT	218	228
			**R**	GCTTCCATACATTTGCTTTTCC		
D4Mit196	4	12.00	**F**	TTGACTGGTCTTATATATCTCTATCCC	306	272
			**R**	TATATTAAATGCTAACTGCTAAGCACA		
D4Mit81	4	38.0	**F**	GACCTGAGTCTTTCTTAAGATGGA	204	160
			**R**	GCTTGCTTTTTTGGCTTCTG		
D4Mit203	4	60.0	**F**	GAATTCTTCCTGGGCCTTTC	111	101
			**R**	CAAGAGCCCAGGTGTGGTAT		
D4Mit226	4	78.50	**F**	CCCCCCAAAAAAAAGAGAAA	115	123
			**R**	AGGATTAGTGAAGGCACTGAGG		
D5Mit352	5	20.0	**F**	CCCAGAGCCCACATCAAG	120	113
			**R**	TAGGTGGGTGTGTCTCTCCC		
D5Mit11	5	26.0	**F**	GATCTTCCTACCTTCTTACCCAC	188	206
			**R**	CATGATTTTATTTGGGGGG		
D5Mit201	5	42.00	**F**	GAGGACTCCTTCGATTTCCC	118	110
			**R**	TTCCTAAGCAGGAACTGACCA		
D5Mit10	5	54.0	**F**	CGAGAAGTTGGAAAGACCCA	188	196

			**R**	GGCACCCATGCCTCTATG		
D5Mit425	5	65.0	**F**	TCGCCTTTCTTTCCCTCC	114	122
			**R**	AAAATTACATTTGCATCTGGGG		
D5Mit99	5	80.0	**F**	CAGAAAAGAGAAAACGGAGGG	204	198
			**R**	TTCCTGCTGCCTGAAGTTTT		
D6Mit139	6	2.50	**F**	ATAGAAGGCGAGAACTAACCCC	119	115
			**R**	TGTTTCTGCCCCTGTAGTTG		
D6Mit74	6	20.50	**F**	CATGTGCAGTGTAAGTAAGACCTC	132	150
			**R**	TCTCCTCCATCCTTCTCCAT		
D6Mit209	6	32.50	**F**	CTCCCCCTCTGTGTGATTGT	128	134
			**R**	TTATTACACCAGACCCATGTGG		
D6Mit61	6	62.30	**F**	TACAGAGGCTAGAACACTCCTGG	146	136
			**R**	CACTTGTTGCTCCTGCTGAG		
D6Mit15	6	74.00	**F**	CACTGACCCTAGCACAGCAG	195	260
			**R**	TCCTGGCTTCCACAGGTACT		
D7Mit191	7	3.40	**F**	TTGGGTTTGTACTACCTAGATACCTC	132	144
			**R**	CCTCTAGGGCTCTTGCACAC		
D7Mit229	7	23.00	**F**	GGTTCTCTTTCCTTGTTTGCC	139	123
			**R**	TACTGGTTACATCTGGTGGGTG		
D7Mit323	7	50.00	**F**	TTTCACCTTCTAATCCTACTTCCTG	106	116
			**R**	TGTCCAGAACAGGAAATAGAGTACC		
D7Mit332	7	65.60	**F**	TACCATCCCTAACTGGTTCTCTT	122	116
			**R**	CTGCACACTCACATACATACTCATG		
D7Mit259	7	72.00	**F**	CCCCTCCTCCTGACCTCTT	152	148
			**R**	GTCTCCATGGGAACCACACT		
D8Mit124	8	6.00	**F**	CAACTGTGTATCATAAACTGGGAA	135	129
			**R**	GAAGAATCACTCAGCAGTGTATGG		
D8Mit24	8	18.00	**F**	ACCTCCACACGATTACAGTGG	168	160
			**R**	ATGATGTCACCAGCAACTTCC		
D8Mit45	8	40.00	**F**	GAACAGGACCAATAAAATGAAAGC	132	122
			**R**	CTACCTTACCAAACTTCCCGG		
D8Mit318	8	57.00	**F**	TTACAGATTTGGGGATTCATATTC	118	124
			**R**	CAGACTCCCAGGTAAAGGCA		
D8Mit280	8	72.00	**F**	CATGCAATTCCAATGTCAGTG	160	108
			**R**	TAGCACTCAATCAAACCCCC		
D9Mit297	9	15.00	**F**	TGCCTCTCTCATCTCTTTAAACA	112	104
			**R**	ATTTGTCTCTCTGTCTTTTGCTCA		
D9Mit97	9	29.00	**F**	TCTCACTACTGCCTGCCAGA	162	154
			**R**	TAGATTTCTCAGGCAAGGAAGC		
D9Mit74	9	41.00	**F**	GAGTCAATCACTGCTGGAATACC	144	136
			**R**	ATGATTGATTGATTGATTGACTGA		
D9Mit355	9	53.00	**F**	CTCATTCACTTCCTGGTCCTG	151	121
			**R**	GAAGGAAAGCCCACACTTTG		
D9Mit151	9	72.00	**F**	TGGTCAAGGTGTGGTATCGA	110	116
			**R**	AAAACTCAGCATCCAATGGG		
D10Mit213	10	11.00	**F**	CTCCTCCTACTGATTGTCCCC	136	150
			**R**	GGGACAAACTTTTAAAAATTGCA		
D10Mit44	10	27.00	**F**	CCACCTTAGACAGTTTACATGGC	160	122
			**R**	CCACGCCCAGCTTACTTCT		
D10Mit31	10	36.00	**F**	CATAAGGAGCACAGGCATGA	154	148
			**R**	CCCTCTACGTGCATGCTGTA		
D10Mit230	10	49.00	**F**	AGATAGCCTAGGGGGTGCAT	142	116
			**R**	ATCAGTTTCCAATCGCTGCT		
D10Mit233	10	62.00	**F**	GTGCTTTATATTGGAGATCATCACA	110	130
			**R**	GTCCCGAATTTCACATACATAGC		
D11Mit226	11	1.55	**F**	AGGTGAACTCTTTTGAAGTTTGTG	126	142
			**R**	AAAGGAGTGACTGAGAAAGACACC		
D11Mit51	11	18.00	**F**	CCAAACAGGGTCTGTTTTATTC	132	140

			**R**	TAACAGGGTGAGTTTAGTGAAACA		
D11Mit278	11	40.00	**F**	AGGTCCTCCCAGATCCTCTC	138	115
			**R**	TATCAACCACTCCAGGACAGG		
D11Mit289	11	55.00	**F**	CTTTGGTTGGTTTTAAATGTTTTAA	119	126
			**R**	AAGGAGAAAGCAGATTCATACACA		
D11Mit214	11	70.00	**F**	CATACAGCCTTCAACAATGACA	135	149
			**R**	ACTGCATACATGTGCACTCATG		
D12Mit56	12	3.00	**F**	GCTGTTTCACAGTCATTCATAACA	156	148
			**R**	AACCTGCACAGGGTTTCCTT		
D12Mit124	12	13.00	**F**	GAGAGTCTCTTAAATCACAAAAATGTG	124	144
			**R**	TGCTCAAAGTGCTGGATGAA		
D12Mit225	12	29.00	**F**	GTTAGCCAAAGCCAAATGGA	116	106
			**R**	CACAATAAAATAAGCAGCAACCC		
D12Mit118	12	45.00	**F**	CATCTTCAATAAAATGGAGATGTACA	124	134
			**R**	CGCTTTCCCTTCATGTACTAGC		
D12Mit8	12	58.00	**F**	TTGCCTAACCCACTCACACC	146	168
			**R**	TGGTGACTCCTTACAGAGGC		
D13Mit88	13	21.00	**F**	ACTGATGGCTCATGAGACCC	182	170
			**R**	AAAATTAATAGGAACTGCAAGGG		
D13Mit54	13	35.00	**F**	CCATGTTTTGAAGCCTGCTT	132	122
			**R**	GACATGGGGAACTGTACCTCA		
D13Mit128	13	48.00	**F**	TTCTAAATATGCATATGACTGCCC	105	113
			**R**	TATGTCATATAACCATTTCAGCATAGA		
D13Mit260	13	65.00	**F**	TAAATTTGGATGCAGACAATGG	109	115
			**R**	TTAAAAATAGAAATGGCTCTGTGTG		
D14Mit60	14	15.00	**F**	AGGCTGCCCATAAAAGGG	112	136
			**R**	GTTTGTGCTAATGTTCTCATCTGG		
D14Mit114	14	30.00	**F**	ATGACATCAGACTTTGGCCC	139	131
			**R**	TTTTGATGTGTATGGATATGTCTCTG		
D14Mit92	14	45.00	**F**	CTGCTGCAGAATTAATTGATTTT	128	90
			**R**	GGATATATGGATTTATACAGACACACA		
D14Mit165	14	52.00	**F**	TGTACATTAAATTTGGAAACCTGG	121	136
			**R**	ACTACACTTTCAGCATAAGACACACA		
D14Mit248	14	60.00	**F**	AAGATGGGTAAAAGCACAGAGC	165	183
			**R**	TTGGTCACCACATGGCTG		
D15Mit53	15	12.30	**F**	CTCCCTTACCTTCGGCTCTT	146	140
			**R**	AGGGTAATTTCAATTAAACTCGTG		
D15Mit107	15	49.00	**F**	CAACACTTATACACTTGTGTCAGGG	145	151
			**R**	TCATGGTTGGAACAGCAGAC		
D15Mit172	15	57.80	**F**	CGGGAAAAGTTTATTTAAAACTGC	134	142
			**R**	GGTCACACATGCATATGTGTATACA		
D15Mit161	15	69.20	**F**	TCTGTTTTGTTTGTTCGTTTGC	106	128
			**R**	TAAAATCTCCCTGTATACAAGTCTGTG		
D15Mit26	15	29.00	**F**	ACAGACTGCTACAAACTTGGAGC	130	112
			**R**	AAATGGCTAAACCCCAGCTT		
D16Mit131	16	4.30	**F**	TGGTGGTGGTGTTGATGGTA	174	144
			**R**	AAGACCATTTCTAATAAACAACACCC		
D16Mit4	16	27.30	**F**	AGTTCCAGGCTACTTGGGGT	147	132
			**R**	GAGCCCTCATTGCAAATCAT		
D16Mit139	16	43.10	**F**	GTATGTAAGGAATGGTCAAATTCTTG	174	148
			**R**	TCATTGTGATTGTGAAAGAATGC		
D16Mit106	16	71.45	**F**	GTTGTTGGTCCACCATACCC	140	148
			**R**	GGGTATGAGGTACAATGCTTTACC		
D16Mit158	16	54.50	**F**	AAGAAGGTGGTGGTTGCTGT	156	140
			**R**	ACTATCCACACCCATCTTAAGAGC		
D17Mit143	17	5.00	**F**	GCTTTCTTGAAGACGTGGGA	132	126
			**R**	CACAGGATGCTTGTAAGCACA		
D17Mit88	17	29.50	**F**	TGGCCTCTGTAGGCATCAG	186	242

			**R**	TGCTATGTAGCAAGTTAGAGGCC		
D17Mit76	17	54.60	**F**	CTCCTCACCCAGATTCTTGTAA	90	124
			**R**	TTTCGCAAGTTATTTTAACCCG		
D17Mit241	17	45.30	**F**	TTTCAAATTCATAGCCTCTTTTTC	132	108
			**R**	TACAGAATGAGCAGGTTGTATTAAGG		
D18Mit110	18	4.00	**F**	GGGAGCCGAATTAACACAAA	121	149
			**R**	AACCAAAAACCTTCAAGATAAACA		
D18Mit14	18	18.00	**F**	GAGGTGATGTGGACACACTC	96	105
			**R**	ACACAGCCTAGAATGCACGG		
D18Mit140	18	37.00	**F**	GGTAGGAGTCACTTTCCGTCC	132	142
			**R**	TTTTGTGAGCATTTTTATACCATT		
D18Mit186	18	45.00	**F**	AAGTGTTGGGCAAAGGCTAA	108	126
			**R**	CTTTAGTATAGTGTGCATGAGTGTGA		
D18Mit53	18	27.00	**F**	TTCCATTGATGCTGCCATTA	130	144
			**R**	GCATAATACCTTATCCTTCGCG		
D19Mit68	19	3.38	**F**	CCAATACAAATCAGACTCAATAGTCG	122	136
			**R**	AGGGTCTCCCCATCTTCCTA		
D19Mit23	19	13.58	**F**	CCATTTCCTTTGAGGATAGTGT	186	196
			**R**	AAATCTTGGCATTTCTCTTACTGG		
D19Mit106	19	20.18	**F**	CCTTTTTTTTTTTAACCAGACAGG	134	124
			**R**	ATCAATGAATGAAGAACAAATAGTTTC		
D19Mit53	19	38.46	**F**	GCACGCCACAACTCAGAG	98	110
			**R**	AGAAAAGGTTCTCCTACCTCTCG		
D19Mit137	19	54.60	**F**	GTCCCTCTTTGTCCCCATTT	132	122
			**R**	TTAATGCTGGTCCTACAAACACC		

Microsatellite markers used in the Genome Wide-Scan study with an average spacing of 20 cM. Amplicon size for each microsatellite are presented for A/J and C57BL/6 strains. Forward and reverse primer sequences are presented for each microsatellite.

Table S2. Designed primers used for fine mapping

**Gene, Protein name**	**Symbols**	**Direction**	**Primer Pairs**
Peroxisome proliferative activated receptor, gamma, coactivator-related 1	*Pprc1*	**F**	AACCTGACTAGCCCAGTCCT
		**R**	AGGACATCAGCTGGAGACAAG
Beta-transducin repeat containing protein	*Btrc*	**F**	GAAGGAAGAGGAGGCGGGAT
		**R**	CTCTGCCGGGTCCATAATCG
Nuclear factor of kappa light polypeptide gene enhancer in B cells 2, p49/p100	*Nfkb2*	**F**	GAGAGAAGCTGTCTGGGTACG
		**R**	GATGCTTAGGGTGTGGGGTT
Sec31 homolog B (S. cerevisiae)	*Sec31b*	**F**	TGGCGTCAGTGTCACATACC
		**R**	TCCAAGGGTCAATCCCTCCC
Progressive external ophthalmoplegia 1	*Peo1*	**F**	GGACGCTCCCACTTCCTTCTGCCTCTTCAT
		**R**	CATGCAAAGAAAGTGTCCTGTGGTCTTGTC
Leucine zipper, putative tumor suppressor 2	*Lzts2*	**F**	CCTTCTTCCGCCAGCAGGATGGGCTGCTAC
		**R**	CAGAGCGGTTCCTGTGCCTCGTAGCCACCC
PDZ domain containing 7	*Pdzd7*	**F**	CCTAGAGCGAAGATGGCCG
		**R**	CCTAGGGTCATTCCGTTCCC
T cell leukemia, homeobox 1	*Tlx1*	**F**	AGCCCATCAGCTTCGGTATC

		**R**	CCAAGGCCGTAGTCTCCATC
Ladybird homeobox homolog 1	*Lbx1*	**F**	CTTCCTCTCGCACCGTCCA
		**R**	CTTGTGCCTACCTTCGGCTG
Polymerase (DNA directed), lambda	*Poll*	**F**	CCCACTCTCCAGGATCTCCATGACCTTCTC
		**R**	TAGCATCCCGGGAATTGGCAAGCGGATGGC

Designed primers covering SNPs on genes selected for fine mapping study, since they are polymorph between background strain A/J and C57BL/6J. Forward and reverse primer sequences are presented for each marker.

Table S3. Microsatellite markers used for haplotyping

**Markers**	**Chr**	**cM**	**Direction**	**Primer Pairs**	**A**	**C57BL/6**
D19Mit106	19	20,18	F	CCTTTTTTTTTTTAACCAGACAGG	134	124
			R	ATCAATGAATGAAGAACAAATAGTTTC		
D19Mit86	19	20.99	F	GTTAGGGCTCTGGAGTTGACC	120	108
			R	ATGAAGTTAAGGAAACTCACAGGC		
D19Mit135	19	26.5	F	GGTTAAAGATGATTGGATGATGG	128	124
			R	TATGCCTGCGTAGGCTTATATG		
D19Mit64	19	28,42	F	AAAAACACATGTGTGTGTCCG	92	96
			R	AATGGAGCAGACCTGAGGG		
D19Mit46	19	29.06	F	ACCCTGCCCTCTCTCTCC	133	115
			R	GCACTCGCCAACTCAGGTAT		
D19Mit63	19	30,30	F	CGCTTTCTTTGACTGGAATG	146	152
			R	GTCCTTTCACTTTCCACATGTG		
D19Mit101	19	32,35	F	GCCAGCCTAGATGCCTATCA	121	125
			R	TTCTTGGAAATCTGATTTTTATTTATG		
D19Mit112	19	36.96	F	ACCTACATTTCTATCCCCCCC	110	108
			R	TGGAGTCCCCCACAGATTT		
D19Mit67	19	37,98	F	GAGAAAAGTTAGCATGCGTGG	115	113
			R	TGGTAAGTAGGCTGTTAGATATGGC		
D19Mit53	19	38,46	F	GCACGCCACAACTCAGAG	98	110
			R	AGAAAAGGTTCTCCTACCTCTCG		
D19Mit9	19	38,97	F	CCGTCTGTATAGTTCCTTTGCC	120	124
			R	CCCTTGTGAGTGCCTGAATT		
D19Mit8	19	39,37	F	CTGTCCAGCCTTTAAGCCAG	172	178
			R	CTAACTGCTTTGGCTCCTGG		
D19Mit83	19	40,41	F	GACACATGCGGCATACAGTC	123	127
			R	CTTGCTCTGAGTATTTTAATGACTGC		
D19Mit91	19	40.53	F	GGGTTGGCTCAATACTCCAA	134	112
			R	CCCCCACCTGGTATCTTGAG		
D19Mit36	19	48,79	F	ATAAGACTTGAGGTTGACCTCTGG	174	164
			R	CTGTAACAATGGGAACTGTAGGG		
D19Mit1	19	50.32	F	AATCCTTGTTCACTCTATCAAGGC	142	121
			R	CATGAAGAGTCCAGTAGAAACCTC		
D19Mit34	19	50.72	F	CAGTGAAAGAACCTGTCGCA	198	156
			R	TTGTATGTGTGCTGAGCATCTG		
D19Mit104	19	51.45	F	CATGTAGTGCACAGACATACATGC	101	106
			R	TCTCATTGCATGTTTAGAGGACA		
D19Mit137	19	54,60	F	GTCCCTCTTTGTCCCCATTT	132	122
			R	TTAATGCTGGTCCTACAAACACC		
D19Mit71	19	56.28	F	ATGATTCCCGCAGTTTTGTC	135	137
			R	TCTCAACTGTTATTCCTCAATAGCC		

Microsatellite markers that differ between background strains A/J and C57BL/6, used in the haplotype study in order to narrow down the QTL region. Primer sequences and amplicon sizes of A/J and C57BL/6 are also presented.

Table S4. PCR primers amplified for Sanger Sequencing

Gene	Direction	Primer Sequence	Amplicon Size
Lbx1	F	CTTCCTCTCGCACCGTCCA	428
	R	CTTGTGCCTACCTTCGGCTG	
Tlx1	F	TTGGGCAAGTCAAGTCTCCA	426
	R	CTAACAGGCAAAACGCTGGC	
Tlx1	F	CTGGACGCACTTCCCTGATT	227
	R	TACTTTGGGGCTTGTGCGAG	
Tlx1	F	CCTTTGGTCCGTCTGTCTGTT	273
	R	CGAGCGAGCCTAGCTGTTTC	
Tlx1	F	AGAGTCAACAGCGAGCGAG	296
	R	CGATACCGAAGCTGATGGGC	
Tlx1	F	ATCGACCAGATCCTCAACAGC	576
	R	TAGTGGGTAGCACAGAGACCG	

Designed primer sequences for *Lbx1* and *Tlx1* covering exons for PCR amplification needed for Sanger sequencing. Primers cover SNPs that are predicted to be polymorph between A/J and C57BL/6 according to Ensembl database.

Table S5. Gene targets for Real-Time PCR

Gene/Protein name	Gene Symbols	Species	Dye Label	Assay ID
Peroxisome proliferative activated receptor, gamma, coactivator-related 1	*Pprc1*	Mouse	FAM	Mm00521078_m1
Nuclear respiratory factor 1	*Nrf1*	Mouse	FAM	Mm01135606_m1
Nuclear factor, erythroid derived 2, like 2	*Nfe2l2, Nrf2*	Mouse	FAM	Mm00477784_m1
Beta-transducin repeat containing protein	*Btrc*	Mouse	FAM	Mm00477687_m1
Nuclear factor of kappa light polypeptide gene enhancer in B cells 2, p49/p100	*Nfkb2*	Mouse	FAM	Mm00479810_g1
Peptidylprolyl isomerase A (Cyclophilin A)	*Ppia*	Mouse	FAM	Mm02342430_g1
Glyceraldehyde-3-phosphate dehydrogenase	*Gapdh*	Mouse	FAM	Mm99999915_g1

Gene targets used in the gene expression analysis, measured with FAM labeled probes. Gene/protein names followed by gene symbols are presented.

Table S6. Potential housekeeping genes tested for Hg exposure

Gene/Protein name	Symbols	Species	Dye Label	Assay ID
Actin beta	*Actb*	Mouse	FAM	Mm02619580_g1
Peptidylprolyl isomerase A (Cyclophilin A)	*Ppia*	Mouse	FAM	Mm02342430_g1
Phosphoglycerate kinase	*Pgk1*	Mouse	FAM	Mm00435617_m1
Eukaryotic 18S ribosomal RNA	*18s*	Mouse	FAM	Mm04277571_s1
Glyceraldehyde-3-phosphate dehydrogenase	*Gapdh*	Mouse	FAM	Mm99999915_g1
Beta-2-microglobulin	*B2m*	Mouse	FAM	Mm00437762_m1
Transferrin receptor (P90, CD71)	*Tfrc*	Mouse	FAM	Mm00441941_m1
TATA box–binding protein	*Tbp*	Mouse	FAM	Mm01277042_m1
Hypoxanthine Phosphoribosyltransferase	*Hprt*	Mouse	FAM	Mm03024075_m1
Tyrosine 3-Monooxygenase/Tryptophan 5-Monooxygenase Activation Protein	*Ywhaz*	Mouse	FAM	Mm03950126_s1

Ten genes selected as potential endogenous controls when exposed for inorganic mercury (2.0 mg Hg/L), measured with FAM labeled probes. Selection of endogenous controls were determined with Ct value and normfinder. The criterion for selection was based on minimal fluctuation of Ct values between samples in order to assume being independent of Hg exposure.

Table S7. Housekeeping genes selected for Hg exposure

	***Gapdh* (Ct)**	***Ppia* (Ct)**	**Geometric mean (Ct)**
**A.SW Males**			
Mean	17,427	19,368	**18,371**
Standard deviation	0,1702	0,307	**0,206**
**A.SW Females**			
Mean	16,061	18,893	**17,419**
Standard deviation	0,530	0,457	**0,475**
**B10.S Male**			
Mean	19,228	21,554	**20,358**
Standard deviation	0,338	0,283	**0,299**
**B10.S Female**			
Mean	18,180	20,597	**19,350**
Standard deviation	0,426	0,405	**0,414**

Gapdh and *Ppia* were selected as housekeeping genes after evaluation of 10 potential endogenous controls in Supplemental Material, Table S6. Ct, geometric mean Ct and Standard Deviation of Ct values for each group of mice are presented. Ct variation for housekeeping genes and their geometric mean is below 1 Ct.

Table S8. Codons and amino acids of SNPs on *Pprc1*

**Class**	**ID (Ensembl)**	**Transcript codon/amino acid A**		**Transcript codon/amino acid C57BL/6**	
SNP[1]	rs30400427	**G**AA	Glu	**A**AA	Lys
SNP[2]	rs30815571	**A**AT	Asn	**G**AT	Asp
SNP[3]	rs30566249	**T**CA	Ser	**C**CA	Pro
SNP[4]	rs30507907	C**C**T	Pro	C**T**T	Leu
SNP[5]	rs30750332	**G**TT	Val	**A**TT	Lle
SNP[6]	rs30360955	**T**CT	Ser	**C**CT	Pro
SNP[7]	rs30352970	**A**GT	Ser	**G**GT	Gly

Seven SNPs on exon 5 on *Pprc1* between A/J and C57BL/6J. Accession numbers followed by transcript codons and amino acids of the seven non-synonymous SNPs (1-7) on *Pprc1* are presented. Bold character represents the SNP on each codon.

Figure S1. Conserved regions of SNPs 1-6

SNP[1]: rs30400427, A.SW (**G**AA), B10.S (**A**AA)

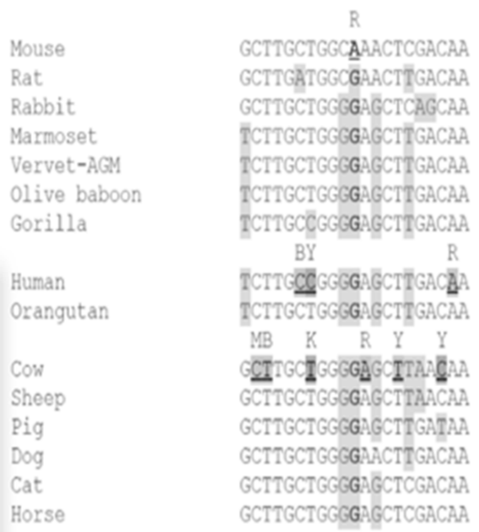

SNP[2]: rs30815571, A.SW (**A**AT), B10.S (**G**AT)

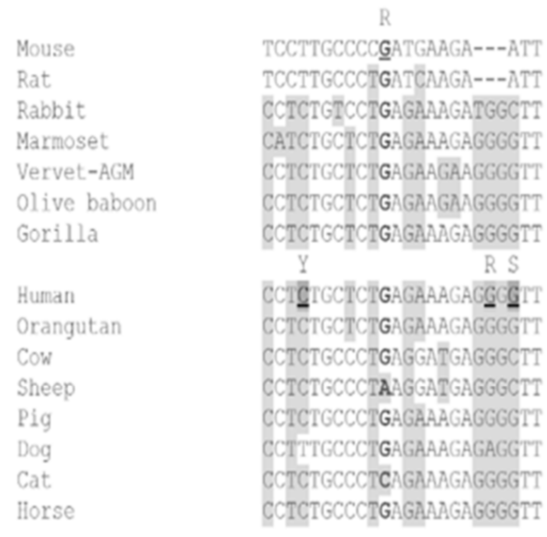

SNP[3]: rs30566249, A.SW (**T**CA), B10.S (**C**CA)

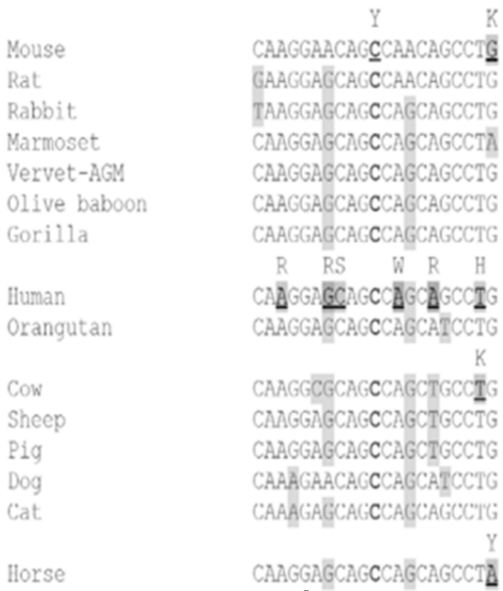

SNP[4]: rs30507907, A.SW (C**C**T), B10.S (C**T**T)

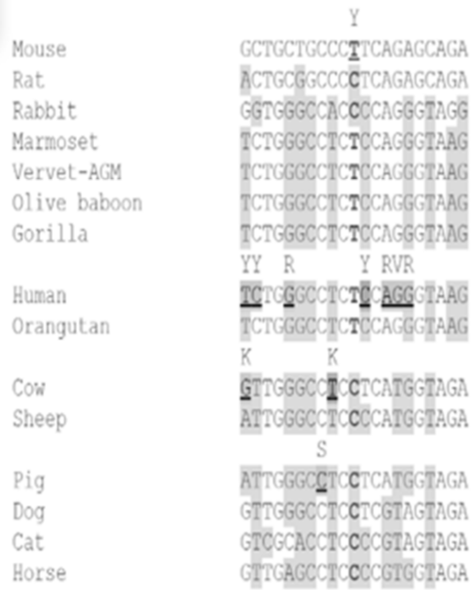

SNP[5]: rs30750332, A.SW (**G**TT), B10.S (**A**TT)

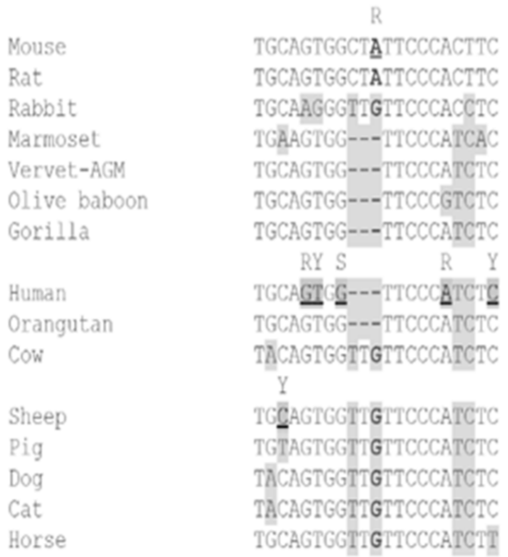

SNP[6]: rs30360955, A.SW (**T**CT), B10.S (**C**CT)

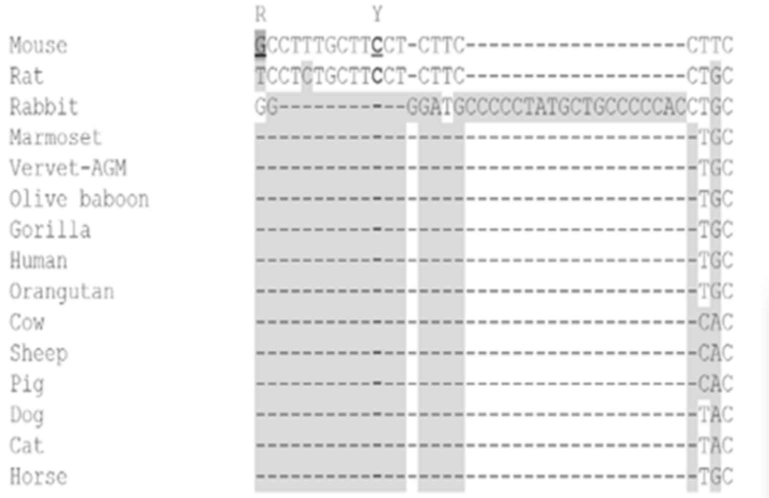

Comparison of conserved regions on 15 mammals on SNPs[1-6] according to Ensembl database. Selection of mammals is due to that Ensembl database have run a nucleotide alignment against them specifically. Accession number followed by codons of A.SW and B10.S are presented in which bold character represents the SNP.

Figure S2. Conserved region of SNP 7.3

SNP[7]: rs30352970, A.SW (**A**GT), B10.S (**G**GT)

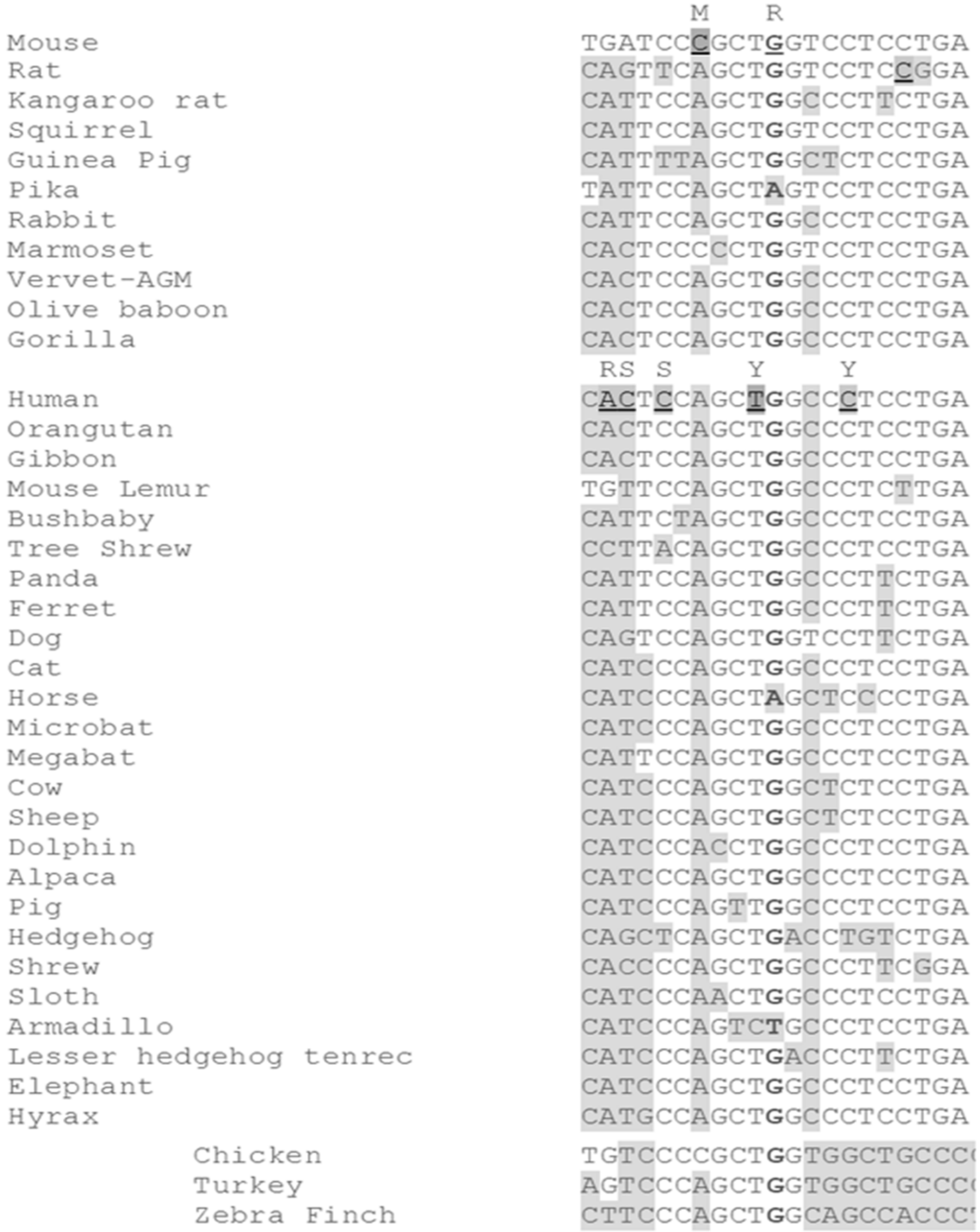

Fifteen mammalian species were selected, in which Ensembl database have run a nucleotide alignment against on rs30352970 in *Pprc1*. Sequence data of all 15 mammals were obtained by using Ensembl database for retrieval of amino acid sequences. Conserved region on amino acids were performed by aligning the multiple sequences with the use of Clustal X (version 2.1).

Figure S3. Amino acid alignment of SNP[7] in *Pprc1*

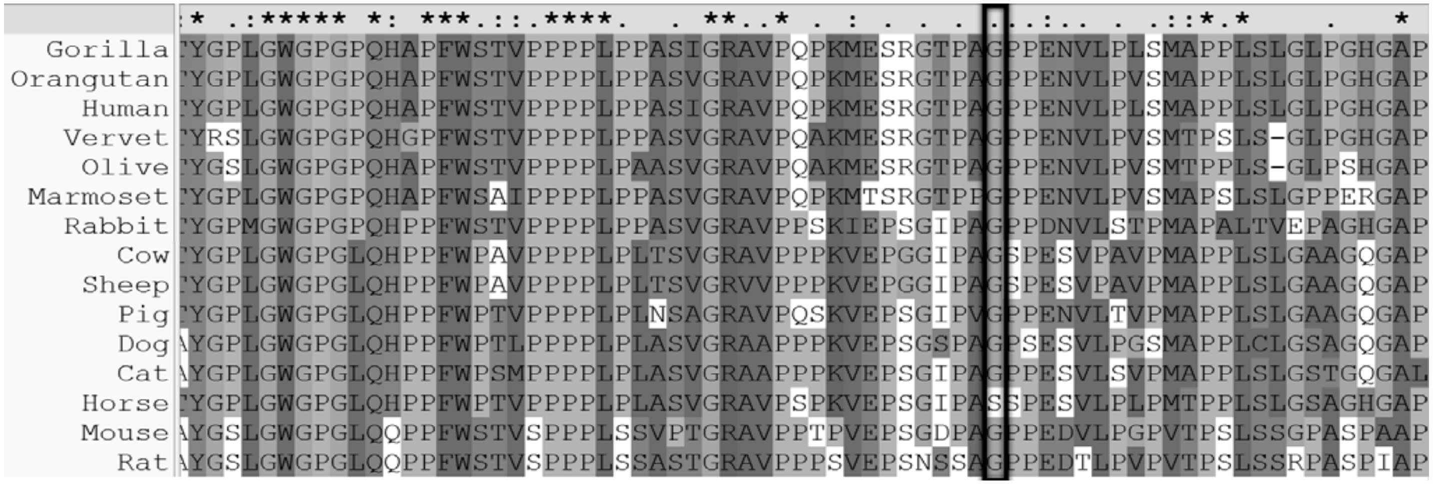

Comparison of conserved region on 36 mammals and three birds, of SNP[7] (rs30352970) according to Ensembl database. Selection of animals is due to that Ensembl database have run a nucleotide alignment against them specifically. Accession number followed by codons of A.SW and B10.S are presented in which bold character represents the SNP.
